# Effect of Educational Intervention on the Fruit and Vegetables Consumption among the Students: Applying Theory of Planned Behavior

**Published:** 2016-10-26

**Authors:** Mohammad Hossein Taghdis, Towhid Babazadeh, Fatemeh Moradi, Fariba Shariat

**Affiliations:** ^a^ Community-Based Participatory Research Center, Iranian Institute for Reduction of High-Risk Behaviors, Tehran, Iran; ^b^ Department of Health Education and Promotion, Student Research Committee, School of Health, Tabriz University of Medical Sciences, Tabriz, Iran; ^c^ Department of Municipal Health in Tehran, Tehran Municipality, Tehran, Iran

**Keywords:** Fruits, Vegetables, Students, Educational intervention, Theory of planned behavior

## Abstract

**Background:** The importance of consuming fruits and vegetables (F&V) in prevention of chronic diseases is known. Childhood play an important role in formation of healthy eating habits. The purpose of this study was to examine the effect of education, with application of the theory of planned behavior, on improvement of F&V consumption.

**Methods:** In this quasi-experimental study, 184 fourth, fifth, and sixth-grade students participated were enrolled from Jan 2013 to Jun 2014. The samples were selected from 6 schools in Chalderan County, West Azerbaijan, Iran through cluster random sampling method. Two out of 6 schools were randomly selected and each was employed in either experimental or control group. The data collection instruments included a researcher-made questionnaire and a 24-h F&V recall. Data were collected after verification of the reliability and validity of the questionnaire.

**Results:** Before the intervention, no significant difference was observed between the intervention and control group regarding attitude, subjective norms, perceived behavioral control, behavioral intention and fruits and vegetables consumption (*P*>0.05). However, after the educational intervention, the mean scores of attitude, subjective norms, perceived behavioral control, behavioral intention variables and fruits and vegetables were significantly higher in the intervention group when compared to the control group(*P*<0.05).

**Conclusions:** Increased behavioral intention, attitude, subjective norms, and perceived behavioral control can promote F&V consumption among the students.

## Introduction

Dietary disorders or deficiency diet in children can be decreased their physical and mental growth, and learning ability. Appropriate diet not only leads to effective learning in children, but has a part in modification of their abnormal behaviors^1^. Fruits and vegetables (F&V) are important component‏ of a appropriate diet. They contain vital elements for health including vitamins, minerals, ﬁbre, and other minor components such as phytochemicals which are potentially beneﬁcial for physical health^2^. A F&V enriched diet is regarded as an important factor in preventing from chronic diseases such as colon cancer and type 2 diabetes ^3-5^.

WHO recommended the intake of a minimum of 400 g/d of F&V in children older than 2 yr for the prevention of chronic diseases^6^. However, a large proportion of population among children and youngsters does not receive the recommended amount in the worldwide^7-10^. For example, the average daily intake of F&V for children in the UK is around 2.8 servings per day ^11^ and in Iran approximately 2.2 servings per day that considerably is lower than the recommended five portions per day^12^. With increasing the intake of F&V to the minimum recommended by the WHO, the incidence of ischemic heart diseases (31%), esophageal cancer (20%) and stroke (19%) can be prevented ^5^.

Factors such as low social and economic situation,‏ price and nutritional awareness of parents and school authorities are impacting F&V consumption ^13,14^. In addition, school can play an important role in promoting healthy eating patterns among children^15^. Appropriate diet‏ education at the elementary school level helps students to learn healthy eating behaviors in the early years of their lives, with the aim of having them continue these behaviors into adulthood^16^. School environment delivers chances to reach children with important dietary messages and reinforce appropriate eating patterns, allowing children to improve appropriate skills for making informed food selections^17^.

The effect of an educational intervention depends on the appropriate apply of behavioral scientific theories^18^. Theory of Planned Behaviors (TPB) is one of the more important theories to design evidence-based interventions^19^. The TPB assumes that attitude, subjective norms, and perceived behavioral control lead to the development of a behavioral intention, and that behavioral intention is assumed to be the immediate antecedent of behaviour^2,20^.

The impact of the different components of the TPB in predicting F&V consumption, and have suggested that the TPB can be applied to improve F&V consumption^2,21^. Behavioral control, attitudes and subjective norms are most important factors in explaining individual's intention to F&V concumption^2^. Moreover, perceived behavioral control is the strongest predicting factor of intention and behavior to explain the healthy nutritional behaviors and in these studies has been recommended the development of educational interventions based on TPB,^21^.

The aim of this study was to examine the effect an educational intervention based on TPB on fruits and vegetables consumption in the male student of elementary schools.

## Methods

### Design and participant

This quasi-randomized controlled trial study was performed from Jan 2013 to Jun 2014 in Chalderan County, west Azerbaijan Province, Iran. Overall, 184 students were selected through sampling method of cluster random. Among the 6 elementary school of the county, two schools were selected randomly, one were randomly selected as the control and intervention group. Then, all of the students of the fourth, fifth, and sixth-grade were included into the study. Finally, the number of people in the experimental and control group was 94 and 90, respectively.

The main criteria of inclusion in the study were students in fourth grade; fifth and sixth grade; students and parents consent to participate in the study. Exclusion criteria were consumption more than 5 serving/per day of fruit and vegetables; lack of consent from study.

### Instruments

In order to collect data, the researcher-made questionnaire based on TBP containing demographic characteristics and 24-h F&V recall questions was used. Intervention and control groups completed the questionnaires before the intervention. According to the findings of pre-test of groups, an educational intervention was designed for students in the intervention group. Finally, both groups were followed 3 months after the intervention.

The researcher-made questionnaire was prepared by reviewing other questionnaires applied in similar studies. Then, validity of the mentioned questionnaire was investigated by an expert panel (include 2 nutritionist and 4 health educationist). The researcher-made questionnaire was given to the expert panel and they were asked to provide their ideas through choosing the options of not related, weakly related, related, and strongly related. According to the specialists recommendations data collection instruments were modified and prepared. In order to assess the reliability, a pilot study performed on 20 students not included in the final sample. The Cronbach’s α coefficient of attitude (0.77); subjective norms (0.80); Perceived behavioral control (0.85) and behavioral intention (0.86) were all at the favorable level.

The theory-based questionnaire is divided into four sections; Behavioral intention (4 questions i.e. I am going to eat at least 5 servings/day of fruits and vegetables at four the next week); Attitude (5 questions i.e. I believe that if I take 5 servings/day fruits and vegetables, remain healthy); Subjective norms (4 questions i.e. My parents are expecting that I take 5 servings/day fruits and vegetables); and perceived behavioral control (7 questions i.e. It is difficult for me at the time of the exams that I take five servings/day of fruits and vegetables). Liker scale (1 to 3) has been used for scoring data collection instruments. Minimum and maximum values of 4-12, 5-15, 4-12, and 7-21 were, respectively, assigned to behavioral intention, attitude, subjective norms, and perceived behavioral control.

In order to investigate how F&V proportion was received, the 24-h F&V recall questionnaire was employed. In this instrument, the amount of F&V consumption was investigated for three consecutive days (including two workdays). Before pre-testing, research's objectives were explained to the students and the informed consent letters were delivered to their parents to not only explain the research's objectives to them, but also invite them to participate in the study. In addition, consent of the fourth, fifth, and sixth-grade teachers for participation in the study was gained. Then, the 24-h F&V recall questionnaires were distributed among the students and their parents, and then, after a full explanation of how to answer the questions was given, completed by the students with parental supervision.

### Developed educational intervention

The educational intervention was developed based on results of the two groups’ pre-tests ^22^.‏ After analyzing data, limitation, needs, and strengths and weaknesses in different fields and then type of content, teaching methods, time and the number of training sessions were designed.

The educational intervention included four 45-min training sessions for the intervention group and one 60-min training session for teachers and parents. The purpose of the training session for teachers and parents was to increasing the subjective norms of the student about fruits and vegetables. At the end of training session, an instructional booklet was given to the participants. In the first and second training sessions for students, participants were instructed about types of food groups, units of fruits and vegetables consumption and its consumption important on the people health. The third training session was held with the aim of improve the attitude of the students. The purpose of the third session was to increase the perceived behavioral control structure. In this session, discussions on the barriers of fruits and vegetables consumption, the students were taught on ways of overcoming barriers of fruits and vegetables consumption. Training materials and instruments included slide, food pyramid poster, educational booklet, and pamphlet.

### Data analysis

Statistical software package (SPSS Inc., Chicago, IL, USA) was utilized. The homogeneity of the baseline data in demographic characteristics and TPB variables of the two groups was analyzed using chi-square and independent *t*-test. Besides, using the Kolmogorov-Smirnov test normality of the data was examined. The paired *t*-test was used to study the changes before and after educational intervention and independent *t*-test to examine the mean of changes and compare the mean of studied variables in both groups. The significance level of *P*<0.05 was taken.

### Ethical Considerations

The study was confirmed by the Ethics Committee of Tehran University of Medical Sciences (approval number 2013.d.130.896) and written informed consent was obtained from all the parents.

## Results

This study was performed on 184 fourth, fifth, and sixth-grade elementary school students (94 and 90 intervention and control students, respectively). Among them, 29.9%, 35.3%, and 34.8% were from the fourth, fifth, a sixth-grade students, respectively. The groups were insignificantly different in terms of demographic data including grade, parental level of education, and parental employment status. Based on the results from the independent *t*-test, no significant difference was observed between the groups with regards to the mean scores of attitude, behavioral intention, perceived behavioral control, and subjective norms before the intervention; while, the mean of changes in attitude, behavioral intention, perceived behavioral control, and subjective norms scores were significant in the intervention group, but not in the control group (Table 1).

**Table 1:** Comparison of TPB Variables before and after the educational intervention in the intervention (n=94) and the control group (n=90)

**Variables**	**Intervention group**		**Control group**		***P *** **value**
	**Mean**	**SD**	**Mean**	**SD**	
Attitude					
Before	9.33	1.87	9.37	1.57	0.884
After	10.61	1.57	1.89	9.67	0.001
*P* value	0.001		0.210		
Subjective norms					
Before	11.54	0.76	10.13	3.03	0.383
After	12.71	4.21	10.83	51.10	0.001
*P* value	0.001		0.530		
Perceived behavioral control				
Before	18.57	2.45	19.04	2.13	0.168
After	19.95	1.15	19.37	2.23	0.029
*P* value	0.002		0.260		
Behavioral intention				
Before	8.08	2.31	8.34	1.68	0.236
After	9.17	2.79	8.43	1.73	0.032
*P* value	0.006		0.140		

Before intervention, no significant difference was seen between intervention and control group with respect to the F&V consumption. After the educational intervention, the fruits diet in the intervention group increased to the average of 0.60±0.65 servings/day, which was a significant change (Table 2). The fruits diet increased to the average of 0.40±0.17 servings/day, but it was not a significant change. In addition, the average daily serving of vegetables diet in the intervention group increased 0.37 ±0.49 serving/day, which was a significant increase (*P*<0.001). However, that serving decreased to 0.10 ±0.23 in the control group (*P*=0.730). In terms of overall F&V intake, it increased to the average of 0.49 ±0.40 servings/day in the intervention group.

**Table 2:** The comparison of F & V consumption before and after the educational intervention in the intervention (n=94) and the control group (n=90)

**Variables**	**Intervention group**		**Control group**		***P *** **value**
	**Mean**	**SD**	**Mean**	**SD**	
Fruits (serving/day)				
Before	0.43	0.27	0.44	0.25	0.547
After	1.03	0.60	0.48	0.25	0.001
*P* value	0.001		0.230		
Vegetables (serving/day)				
Before	0.48	0.27	0.46	0.26	0.337
After	0.85	0.23	0.45	0.24	0.001
*P* value	0.001	0.650		
Fruits and vegetables				
Before	0.46	0.23	0.44	0.22	0.170
After	0.95	0.30	0.47	0.16	0.001
*P* value	0.001		0.094		

## Discussion

This is the first study conducted to promote F&V consumption in students of the male’ elementary schools in Chalderan County, West Azaerbaijan Province. The findings indicated increased F&V consumption among the intervention students. In the present study, F&V consumption rates have increased 0.6 and 0.37 servings/day in the intervention group, after the educational intervention. The effect of educational intervention on improvement of F&V consumption in the fourth-grade primary students in Isfahan is reported^23^. The F&V consumption increased 0.37 and 0.75 servings/day in the intervention group, after educational intervention. In addition, F&V consumption increased 0.49 servings/day in the intervention group, three months after intervention^24^. The educational intervention increase F&V consumptions rates to 0.8 and 0.3 servings/day in the children aged 5-12 year^25^. Training resulted into increase in F&V consumption to 0.52 and 0.24 servings/day in the students^26^. School environment is a suitable place to formation of appropriate eating habits for students. Thus, it is recommended that educational programs in schools for students in order to F&V improvement be taken place.

The results from this study indicate that F&V intake increased (0.49 servings/day) in the intervention group, three months after intervention. The positive impact of education intervention on F&V consumption has been confirmed in some studies^25,27^. The results from educational interventions to increase F&V consumption were different in students. Among the probable reasons of such differences in the results of educational intervention is educational training methods, economic, social, and cultural varieties in the target groups, and data collection techniques used in different studies.

Attitude as a mental process is regarded as the determinant of actual and potential actions. Having positive attitude toward the behavior resulted into the facilitation of that behavior. Making positive attitude is among the methods used in educational intervention to motivate a behavior. In the present study, group discussion and Q&A sessions were held to create positive attitude towards F&V consumption. Using group discussion methods, which stimulate people's mind, broaden their mental skills, and engage them in active participation in learning, can create positive attitude towards healthy eating in the grades 4 and 5 primary students^28^. The results of the our study indicated a statistically significant difference between the intervention group’s attitude scores before and after the educational intervention, which was in agreement with the findings of other studies^23, 29, 30^. Therefore, it is recommended to emphasize improving attitude towards the behavior in educational interventions to increase intake of F&V in students.

Among the other constructs of the theory of planned behavior, used in behavior prediction, are subjective norms. The subjective norms are related to the perception of social pressures on an individual to perform a task. Subjective norms are regulating the standards, either accepted or rejected by people. In this study, the subjective norms have been investigated from parental, teacher, and peer perspectives. The research findings indicate significant changes in the subjective norms after educational intervention. The findings of this study confirmed that engaging the parents, teachers, and peers in educational programs could increase social support^29, 31^. For example, in the previous study, social support through increasing the access to fruit and vegetable can improve students' F&V consumption^32^. Moreover, in educational intervention should consider subjective norms as an important factor in the behavior changes.

The perceived behavioral control refers to one's voluntary control over doing or not doing a task. Even when one's attitude and subjective norms are strong but has no voluntary control over the behavior, he or she may not perform that behavior. In fact, the perceived behavioral control indicates one's perceptions on the existence of advantages and opportunities. In the present study, students' perceived behavioral controls were significantly changed after intervention. These findings are in accordance with studies^29,33^. These results show that belief in control of a behavior could lead to a better performance in individual. Besides, considering the perceived behavioral control is important in educational interventions.

Another construct of the theory of planned behavior, used in this educational program, is behavioral intention. In this theory, the behavioral intention is regarded as the most important behavior predictor. Intentions act as motivational factors that are affecting behavior and indicating the intensity of individuals' attempts to perform a task. The more strong behavioral intention is, the more probable is to perform a behavior. However, there is no 100% relation between intention and behavior; in fact, intention is required for behavior but is not sufficient.‏ Educational intervention could positively affect the students' behavioral intention. The results of the present study are in accordance with those of the previous studies^29,34^ and highlight the effectiveness of the TPB structures in persuasion the student to consumption of F&V. TPB assumes that behavior happens after a behavioral intention; therefore, without behavioral intention, no behavior is demonstrated ^35^. The higher one’s intention score, the more likely one will be to show a certain behavior and display a higher tendency to performance that behavior. Furthermore, based on TPB, behavioral intention is the most nearest predictor of behavior, with attitudes, perceived behavioral control and subjective norms influencing behavior through intentions^35^. Therefore, TPB is a suitable theoretical framework for considering predictive factors of intention of students for F&V consumption.

One of the limitations of the present study was that the data were gathered by a self-report tool. The study was performed on male students only, so it is not possible to compare the results among both males and females. Besides, follow-up was only 3 months after the intervention and not followed up in long periods of time for the durable assessing.

## Conclusions

The outputs of the present study demonstrate the effectiveness of planned behavior theory-based educational intervention in F&V consumption of primary students. Making such interventions in schools can form safe nutrition habits in childhood, which in its turn can contribute to healthy lifestyle in adulthood. In addition, safe and sound nutrition education in the schools can increase educational growth and learning, yield of educational investment, and finally national productivity. Regarding that in this study students’ F&V intake was lower than the recommended level, both before and after intervention, so an urgent attention should be given to the quality of students' nutrition. This requires further investment in educating parents and society, as well as performing more investigations.

## Acknowledgments

This article, as a part of research plan (#92-02-62-20884), approved by Health Research Board of Tehran Medical Sciences University, is the result of an MSc thesis in Health Education. Here, we are grateful to the Chalderan's Instruction and Education authorities for their cooperation and support, and to the teachers and students that participated in this study.

## Conflict of interest statement

The authors declare that there is no conflict of interest.

## Highlights

- The intervention increased consumption of F&V in the students.
- F&V intake was lower than the recommended level in student.
- TPB can be provided a suitable framework for developing educational interventions regarding F&V intake in student.
